# Peripheral proinflammatory Th1/Th17 immune cell shift is linked to disease severity in amyotrophic lateral sclerosis

**DOI:** 10.1038/s41598-020-62756-8

**Published:** 2020-04-03

**Authors:** Mengmeng Jin, Rene Günther, Katja Akgün, Andreas Hermann, Tjalf Ziemssen

**Affiliations:** 1 Department of Neurology, Technische Universität Dresden, Dresden, Germany; 2 Center for Clinical Neuroscience, University Hospital Carl Gustav Carus, Dresden, Germany; 3 German Center for Neurodegenerative Diseases (DZNE), Dresden, Germany; 4 Translational Neurodegeneration Section “Albrecht-Kossel”, Department of Neurology, University Medical Center Rostock, University of Rostock, Rostock, Germany; 5 German Center for Neurodegenerative Diseases (DZNE) Rostock/Greifswald, Rostock, Germany

**Keywords:** Prognostic markers, Amyotrophic lateral sclerosis, Chronic inflammation

## Abstract

Neuroinflammation is involved in the pathogenesis of amyotrophic lateral sclerosis (ALS), but only limited data are available on systematic peripheral and central immune cell profiles in ALS. We studied detailed immune profiles of 73 ALS patients and 48 healthy controls (controls) in peripheral blood by fluorescence-activated cell sorting as well as cytokine expression profiles in serum. In a subgroup of 16 ALS patients and 10 controls we additionally studied cerebrospinal fluid (CSF) samples. In peripheral blood, T cell subtypes presented a shift towards pro-inflammatory Th 1 and Th 17 cells whereas anti-inflammatory Th2 and T regulatory cells were decreased. Important players in innate immunity including distinct monocyte (Mo) and natural killer (NK) cell subtypes were changed in ALS patients compared to controls. Pro-inflammatory serum cytokines such as interleukin (IL)-1 beta, IL-6 and interferon-gamma (IFN-gamma) were increased and the anti-inflammatory cytokine IL-10 was decreased. Correlation analysis revealed moderate negative correlations between Th1 and Th17 to the ALS functional rating scale revised (ALSFRS-R) and to forced vital capacity. In CSF samples, no relevant alteration of the immune profile was found. In conclusion, the immune profile in ALS was shifted towards a Th1/Th17 cell-mediated pro-inflammatory immune response and correlated to disease severity and progression. Large prospective studies are needed to confirm these findings.

## Introduction

Amyotrophic lateral sclerosis (ALS) is a devastating neurodegenerative disease, which affects the upper and lower motoneurons (MN) leading to progressive muscle weakness, paralysis and ultimately death due to respiratory failure. The pathophysiological mechanisms are still unresolved, although increasing knowledge gained in the last two decades. Multiple studies corroborated that numerous pathological processes are involved in MN degeneration and neuroinflammation gained increasing attention and had deemed as a pathological hallmark of ALS^1–3^.

It is commonly accepted that the non-neuronal neighbors play a significant role in the degenerative process of this vulnerable cell type^4^. One of the most impressive pathohistological characteristics during disease progression is activation and increase abundance of microglial cells in the central nervous system (CNS) shown in animal models of ALS^5^ as well as in human studies^6–8^.

Microglial cells are considered to be the resident mononuclear phagocytes in the CNS and can be renewed by bone marrow cells under special circumstances^9,10^. It is supposable that specialized peripheral immune cell subsets are attracted to the central compartment to contribute and perpetuate the pathological processes in ALS disease by local inflammation or activation of resident cells. Therefore, neuroinflammation is not limited to the CNS, it needs a systemic inflammatory response to enable the local inflammation. In general, a systemic inflammatory response is very complex, a plethora of different immune cells are involved, diverse cytokines are secreted and the process is highly dynamic. In ALS, first studies exist on systemic immune response, however contradictory results for different cytokines and immune cells were published^11–19^. While elevated blood and CSF levels of tumor necrosis factor-alpha (TNF-α), interleukin (IL)-17 and interferon (IFN)-gamma were found by independent groups^12–16^, contradictory results were reported for IL-6, IL-1 beta and IL-10^13,16–19^. Inconsistent results were also published concerning changes in the adaptive and innate immune system in the blood of ALS patients, including helper/inducer (CD4+) T cells and cytotoxic/suppressor (CD8+) T cells, monocytes (Mo) and B cells^20–23^.

So far it has been difficult to draw a conclusive picture about the involvement of the systemic immune response in ALS. One reason might have been that ALS pathogenesis progresses fast and thus patients in different cohorts did not represent similar disease stages.

T helper 17 (Th17) cells and regulatory T cells (Treg) are a subset of CD4+ T cells recently caught more attention which play an important role in promoting (Th17) or suppressing (Treg) inflammation and are crucial in the development of different autoimmune diseases, such as multiple sclerosis^3,24,25^. Treg cells were inversely correlated with progression rates in ALS patients and the transfer of endogenous Treg cells of ALS mice from early disease stages into ALS mice lengthen disease duration and prolong survival, suggesting a neuroprotective function of these cells in ALS^26,27^. Th17 cell activation was recently reported in the SOD1 ALS mouse model and this was enhanced after motor nerve injury^24^. One hypothesis is therefore that due to MN injury, MN specific antigens are released and processed by antigen-presenting cells (APC; e.g. monocytes or dendritic cells) inducing priming of specific Th17 cells and their recruitment into the CNS, which promote a MN-specific inflammation leading to MN death^3^. So far only one study was investigating Th17 cells in the blood of ALS patients, showing an increase of this immune cell population in ALS patients^25^.

Thus, alterations of the peripheral immune system in ALS patients need further clarification and changes of single immune cells or cytokines should be interpreted in the context of the entire immune system in ALS and not only as single parameters. Therefore, the aim of this study was to systematically and comprehensively analyze the immunoprofile of peripheral blood mononuclear cells (PBMCs) and CSF in a prospective, unbiased cohort of ALS patients and to determine whether these immunological changes correlate with the disease progression and other disease-related markers.

## Materials and Methods

### Participants and assessments

We collected and analyzed blood and CSF samples in a total group of 73 patients with sporadic ALS according to the revised El Escorial criteria and 48 aged and sex-matched healthy controls (controls) at the motoneuron expertise center Dresden (Department of Neurology, Technische Universität Dresden, Dresden, Germany) from August 2016 to February 2018. Additionally we recorded age, gender, bulbar symptoms, disease subtype at onset, body mass index (BMI), forced vital capacity (FVC), percentage of predicted FVC (ppFVC, is adapted to age, sex and body height for each patient), disease duration (time between the first symptom and the date of sample collection), the ALS Functional Rating Scale revised (ALSFRS-R), ALSFRS-R slope (the decline of ALSFRS-R from symptom onset to the date of sample collection = (48 − score at date of sample collection)/(duration between symptom onset and date of sample collection) and supportive care (invasive ventilation (IV), non-invasive ventilation (NIV) and gastrostomy (PEG)). To check for differences between fast and slow progressors, we defined slow progressors with an ALSFRSR-slope <0.5/month (35 ALS patients) and fast progressors with an ALSFRSR-slope of ≥0.5/month (38 ALS patients) according to Ludolph *et al*.^28^. Patients and controls with any clinical signs of acute infection were not included. Subjects with any history of neurodegenerative diseases were not included in the control group. All participants had not received immune-suppressive therapy. All subjects gave their informed consent and study approval was obtained by the local ethics committee (Ethikkommission an der Technischen Universität Dresden, EK393122012), all methods were performed in accordance with the relevant guidelines and regulations.

**Table 1:** Demographic and clinical characteristics of study populations.

	ALS	controls
Total number (n)	73	48
Sex m/f (%)	42.50/57.50	52.10/47.90
Age in years ± SD	66.65 ± 9.41	63.40 ± 12.10
Onset b/s (n)	21/52	NA
Disease duration in years (Range)	2.48 (0.12–12.02)	NA
Body height in m (Range)	1.69 (1.52–1.88)	NA
Weight in kg (Range)	72.4 (39.0–132.0)	NA
BMI (Range)	25.4 (16.88–40.12)	NA
FVC in ml (Range)	1918 (100–4850)	NA
ppFVC in % (Range)	61.4 (2.53–131.32)	NA
ALSFRS-R (Range)	33 (0–47.0)	NA
ALSFRS-R slope (Range)	0.80 (0.01–3.34)	NA
Supportive Care (IV, NIV, PEG) (n)	17	0
IV (n)	6	0
NIV (n)	10	0
PEG (n)	6	0

n = number; m/f = male/female; b/s = bulbar/spinal; BMI = body mass index; FVC = forced vital capacity; ppFVC = percentage of predicted FVC; ALSFRS-R = Amyotrophic lateral sclerosis functional rating scale Revised; IV = invasive ventilation; NIV = non-invasive ventilation; PEG = percutaneous endoscopic gastrostomy; NA = not applicable.

**Table 2:** Profile of immune cells in CSF of ALS patients and controls.

Immune cells (%)	ALS N = 16 (Mean ± SD)	controls N = 10 (Mean ± SD)	p-value
Lymphocyte	51.59 ± 13.01	39 ± 12.06	0.048*
CD3 + T cells	12.78 ± 17.51	20.74 ± 26.40	0.280
CD4 + T cells	37.89 ± 24.45	41.76 ± 25.77	0.660
CD8 + T cells	16.09 ± 9.39	27.36 ± 19.0	0.140
CD4 + naïve T cells	15.54 ± 7.90	26.08 ± 17.47	0.250
CD4 + memory T cells	20.77 ± 13.85	13.5 ± 8.41	0.130
CD8 + naïve T cells	38.61 ± 20.72	19.52 ± 16.73	0.014*
CD8 + memory T cells	20.84 ± 14.62	21.13 ± 21.12	0.670
B cells	6.93 ± 2.97	11.03 ± 10.28	0.410
Naïve B cells	83.19 ± 11.2	86.34 ± 6.10	0.620
Memory B cells	13.51 ± 5.78	10.44 ± 6.14	0.280
NK cells	9.59 ± 4.77	8.14 ± 3.40	0.410
NK T cells	2.01 ± 1.78	2.14 ± 1.46	0.530
CD4 + NK T cells	25.61 ± 18.81	22.73 ± 25.48	0.340
CD8 + NK T cells	23.41 ± 15.76	37.27 ± 23.79	0.075
Classical Mo	13.28 ± 7.83	15.18 ± 13.06	0.830
Intermediate Mo	8.89 ± 4.27	7.98 ± 5.63	0.670
Nonclassical Mo	7.50 ± 4.52	14.5 ± 13.28	0.220
SlanDCs	4.34 ± 1.98	4.60 ± 2.57	0.920
BDCA1 + DCs	8.69 ± 7.54	6. 9 ± 3.77	0.980

### Preparation of samples

Peripheral blood samples (73 ALS and 48 controls) were collected in lithium-heparin tubes (Sarstedt). PBMCs were prepared by Ficoll–Hypaque (Biochrom) density centrifugation, then washed with phosphate-buffered saline (PBS) and counted manually. Serum samples for cytokine analysis were collected in 7.5 ml tubes (Sarstedt) and centrifuged at 2000g for 10 min, aliquoted and frozen at −80 °C within 20 min after collection.

CSF samples (16 ALS and 10 controls) were centrifuged at 250 g for 10 min at 4 °C within 20 min after collection, the cell pellet was collected and immediately processed by flow cytometry.

### Immune cell phenotyping using Fluorescence-activated cell sorting (FACS)

Freshly prepared PBMCs were used. Subpopulations of T cells, B cells, natural killer (NK) cells and antigen-presenting cells (APC) were characterized by surface staining with fluorescence labelled anti-CD3, anti-CD4, anti-CD5, anti-CD8, anti-CD14, anti-CD16, anti-CD19, anti-CD25, anti-CD27, anti-CD38, anti-CD45RA, anti-CD45RO, anti-CD56 and anti-HLA-DR (BD Bioscience); anti-BDCA1, anti-BDCA2, anti-BDCA3, anti-BDCA4 and anti-slan (Miltenyi Biotec). Negative controls included directly labeled or unlabeled isotype-matched irrelevant antibodies (BD Biosciences). Freshly prepared CSF cells were used directly for FACS analysis. Fluorescence-labeled antibodies for surface staining were used as follows: anti-CD3, anti-CD4, anti-CD8, anti-CD14, anti-CD16, anti-CD19, anti-CD25, anti-CD27, anti-CD45RA, anti-CD45RO, anti-CD56 and anti-HLA-DR (BD Bioscience); anti-BDCA1 and anti-slan (Miltenyi Biotec). All cells were measured on a LSR-Fortessa (BD Biosciences) and evaluated by FACS-Diva Software (BD Bioscience).

### Intracellular staining for FACS analysis

For additional characterization of intracellular markers PBMCs were suspended in culture medium consisting of RPMI 1640 (Biochrom), 5% human AB serum (CC pro), 2 mM L-glutamine, 100 U/ml penicillin and 100 µg/ml streptomycin (Biochrom). PBMCs were stimulated with 10 ng/ml phorbol myristate acetate (PMA, Sigma-Aldrich) and 1 µg/ml ionomycin (Sigma-Aldrich) in the presence of 0.2 µM Monensin (Biomol) for 6 hours prior to analysis. First, cell surface markers were stained as described above. Before the characterization of intracellular markers, cells were fixed with freshly prepared fixation concentrate, and permeabilized with wash-permeabilization concentrate (Fixation/Permeabilisation Buffer Set, eBioscience). Subsequently, cells were stained using fluorescence-labeled anti-IFN-gamma, anti-IL-17A (BioLegend), anti-IL-4 (BD Bioscience), anti-FoxP3 antibody (Miltenyi Biotec) or isotype-matched irrelevant antibody (BD Biosciences) (Suppl. Fig. 6). After the staining procedure, cells were evaluated by flow cytometry. Cells were measured on a LSR-Fortessa (BD Bioscience) and evaluated by FACS-Diva Software (BD Bioscience).

### Cytokine assay

The frozen serum samples and CSF supernatants were thawing one hour before the ELISA measuring was performed. The cytokine concentration was evaluated using commercial ELISA kits for IL-1beta, IL-6, IL-10, IL-12, TNF-alpha, IFN-gamma ELISA kits (BD bioscience), IL-17A and IL-23 ELISA kits (eBioscience) and determined according to the manufacturer’s instructions.

### Statistical analysis

As the samples were not normally distributed by calculation with Shapiro–Wilk test, statistical comparisons of data between two groups were made using the non-parametric Mann–Whitney U-test (MWU). The chi-squared test (χ^2^) was used for a comparison of gender differences between ALS and controls. Spearman rank correlation coefficients were used to examine correlations with a correlation coefficient of r < 0.3 considered as a weak, r = 0.3–0.59 a moderate, and r > 0.6 a strong correlation (modified to^29^). Data were analyzed using the software program GraphPad Prism (GraphPad Software Version 6.0). If not mentioned otherwise, all data are displayed as means ± standard deviation (SD). Significance level was set at p < 0.05. Correction for multiple testing was not applied as the study is merely exploratory, hypothesis-generating, thus a previous hypothesis was not proposed.

## Results

### Demographic and clinical characteristics

Clinical data and bio-samples were collected from 73 ALS (73 blood/16 CSF) patients and 48 controls (39 blood/10 CSF). Demographic and clinical characteristics of the study populations are shown in Table 1. ALS and controls did not differ regarding sex (χ^2^, p = 0.30) and age (MWU, p = 0.14). Disease duration in ALS patients was 2.48 ± 2.35 years, mean ALSFRS-R was 33 ± 11 points and 60% of all cases were classified as spinal onset and 40% as bulbar onset.

### Peripheral immune cell phenotyping in ALS patients

**Figure 1:**
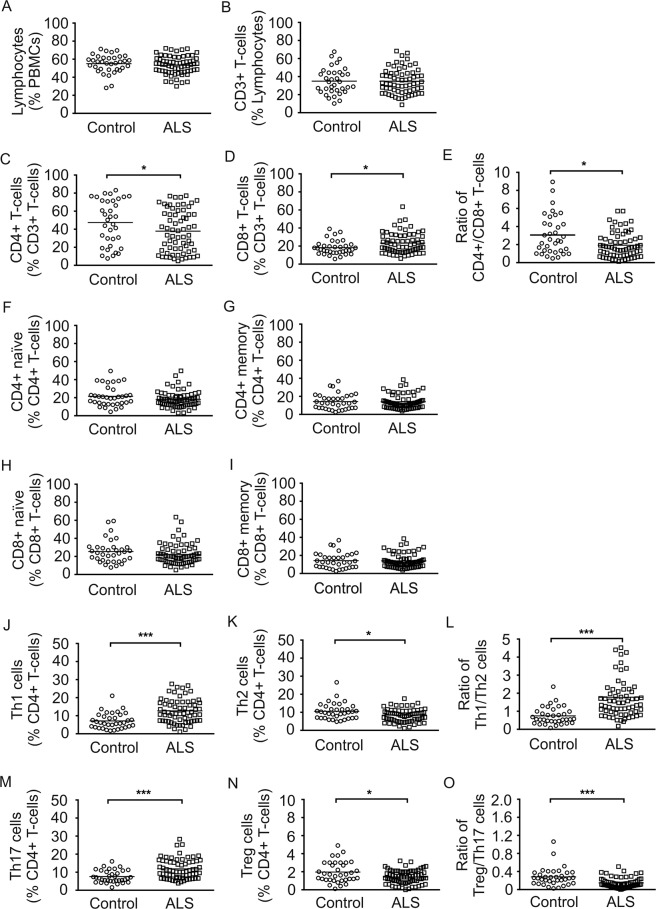
Peripheral T cell profile in ALS patients and healthy controls. Depicted are relative counts of lymphocytes (**A**), CD3 + T cells (**B**), CD4 + T cells (**C**), and CD8 + T cells (**D**), ratio of CD4 + T cells and CD8 + T cells (**E**), naïve CD4 + T cells (**F**), memory CD4 + T (**G**), naïve CD8 + T cells (**H**), memory CD8 + T cell (**I**), Th1 cells (**J**), Th2 cells (**K**), Th1/Th2 ratio (**L**), Th17 cells (**M**), Treg cells (**N**) and the Treg/Th17 cell ratio (**O**) of ALS patients and healthy controls. Results are presented as raw data, each circle represents one healthy control participant and each quad one ALS patient, horizontal line indicate mean values. *p < 0.05, **p < 0.01, ***p < 0.001.

In order to study the alterations of immunoprofiles in the peripheral blood of ALS patients, we performed FACS analysis on PBMCs of ALS patients and healthy age- & sex-matched controls. Lymphocyte subtypes are important players especially in the adaptive immune response. In our study, there was no difference in relative lymphocyte and CD3 + T cells count between ALS and controls (Fig. 1A,B). Subtype analysis revealed a significantly lower relative amount of CD4 + T cells (p < 0.05) and a significantly higher relative amount of CD8 + T cells (p < 0.05) in the ALS group (Fig. 1C,D). Hence, the ratio of CD4 + /CD8 + T cells was significantly reduced (p < 0.05) in the ALS group compared to controls (Fig. 1E). Naïve (CD45RA+) and memory (CD45RO+) CD4 + and CD8 + T cell subsets did not differ between ALS and controls (Fig. 1F–I). In ALS patients, relative count of pro-inflammatory Th1 (CD3 + CD4 + IFN-gamma +) cells significantly increased (p < 0.001) whereas anti-inflammatory Th2 (CD3 + CD4 + IL-4 +) cells significantly decreased (p < 0.05), which led to an increase of Th1/Th2 ratio (p < 0.001) (Fig. 1J–L). Additionally, Th17 cells (CD3 + CD4 + IL-17+) were significantly higher (p < 0.001) in the ALS group and T regulatory (Treg) cells (CD3 + CD4 + CD25 + FoxP3+) were significantly lower (p < 0.05) in comparison to controls. The Treg/Th17 ratio was significantly decreased (p < 0.001) in ALS patients (Fig. 1M–O). We could not find any significant differences for the relative CD19 + B cell count as well as naïve (CD27-CD38-) and memory (CD27 + CD38-) B cell and B regulatory cell (CD5 + CD19+) subtypes (Suppl. Fig. 1A–D).

**Figure 2:**
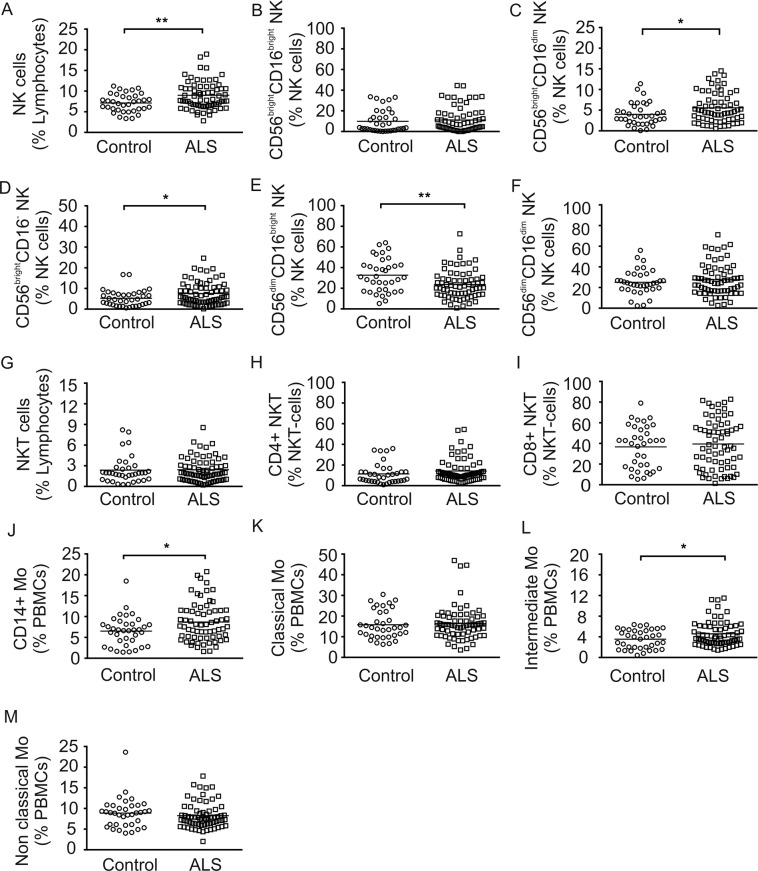
Peripheral NK cell and Mo subsets in ALS patients and healthy controls. Depicted are relative counts of NK cells (**A**), CD56^bright^CD16^bright^NK cells (**B**), CD56^bright^CD16^dim^NK cells (**C**), CD56^bright^CD16^-^NK cells (**D**), CD56^dim^CD16^bright^NK cells (**E**), CD56^dim^CD16^dim^NK cells (**F**), NKT cells (**G**), CD4 + NKT cells (**H**), CD8 + NKT cells (**I**), CD14 + Mo (**J**), classical Mo cells (**K**), intermediate Mo cells (**L**) and non-classical Mo cells (**M**) of ALS patients and healthy controls. Results are presented as raw data, each circle represents one healthy control and each quad represents one ALS patient, horizontal lines indicate mean values. *p < 0.05, **p < 0.01, ***p < 0.001.

NK cells are key players in both innate and adaptive immunity. The relative number of NK cells (CD56 + CD3-) was significantly higher (p < 0.01) in ALS compared to controls (Fig. 2A). Subtype analysis revealed significant higher relative amounts of CD56^bright^CD16^dim^NK cells (p < 0.05), CD56^bright^CD16^-^NK cells (p < 0.05) and significantly lower amounts of CD56^dim^CD16^bright^NK cells (p < 0.01) in ALS (Fig. 2C–E). The CD56^bright^CD16^bright^NK cells, CD56^dim^CD16^dim^NK cells, NKT cells (CD56 + CD3+), CD4 + NKT cells and CD8 + NKT cells did not significantly differ between the two groups (Fig. 2B,F, G–I). Antigen presenting cells (APCs) including monocytes (Mo) and dendritic cells (DCs) are important players of the innate immunity. Total CD14 + monocyte population (Mo) was significantly higher in comparison to healthy controls (Fig. 2J; p < 0.05). Evaluation of Mo subtypes presented increased intermediate (p < 0.05) Mo (CD14 + + CD16+) whereas classical (CD14 + + CD16-) and non-classical Mo (CD14 + CD16 + +) were not altered (Fig. 2K–M). There were no significant differences for the major DC populations including slanDCs (MDC8 + HLA-DR+), BDCA1 + DCs, BDCA2 + DCs, BDCA3 + DCs, BDCA4 + DCs between both groups (Suppl. Fig. 2A–E).

### Peripheral immune cell phenotypes in relation to ALS progression rate

Very recently, much attention has been drawn on the ALS progression rate and its role on treatment response^28,30^. However, not much is known about differences in the progression rate of ALS disease and their contributing factors. Therefore we asked whether we can distinguish slow and fast progressive ALS by immune cell pattern using a cut off of 0.5 points decline per month in the ALSFRS-R score. Interestingly, there were no detectable group differences (Suppl. Fig. 1E–H, Suppl. Fig. 2F-J, Suppl. Figs 3–4), except of a significantly higher amount of CD56^bright^CD16^dim^NK cells in slow ALS compared to fast ALS (p < 0.05) (Suppl. Fig. 4C).

### Peripheral immune cell phenotypes in correlation to clinical characteristics

**Figure 3:**
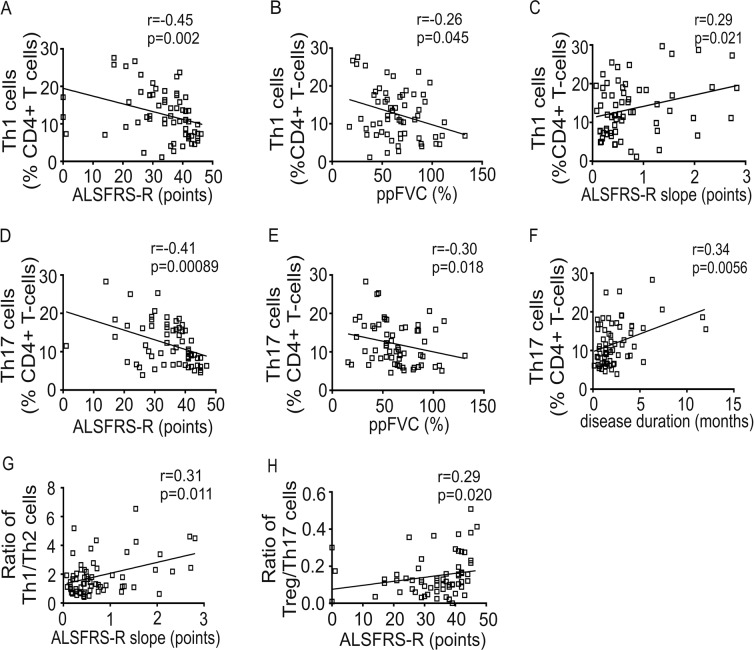
Correlation of T cell subsets to clinical parameters in ALS patients. Correlations of Th1 cells to Amyotrophic Lateral Sclerosis Functional Rating Scale-revised (ALSFRS-R) (**A**), Th1 cells to percentage of predicted forced vital capacity (ppFVC) (**B**), Th1 cells to ALSFRS-R slope (**C**), Th17 cells to ALSFRS-R (**D**), Th17 cells to ppFVC (**E**), Th17 cells to disease duration (**F**), Th1/Th2 ratio to ALSFRS-R slope (**G**) and Treg/Th17 ratio to ALSFRS-R (**H**) are shown as scatterplots. Spearman rank correlation coefficient (r), significance level (p).

To further evaluate coherences to further clinical and demographic parameters (ALSFRS-R, ALSFRS-R slope, disease duration, ppFVC, age and BMI), we calculated spearmen rank correlation coefficients (**Suppl. Tab. 1**). Age and BMI did not correlate to immune cell count, except for CD8 + T cells and CD56^dim^CD16^bright^NK cells. Th1 cells negatively correlated to ALSFRS-R and ppFVC and positively with ALSFRS-R slope (Fig. 3A–C). Th17 cells negatively correlated to ALSFRS-R as well as to ppFVC and positively to disease duration (Fig. 3D–F). The Th1/Th2 ratio correlated positively with ALSFRS-R slope and the Treg/Th17 ratio correlated positively with ALSFRS-R (Fig. 3G,H).

### Cytokine profile in the serum of ALS patients

**Figure 4:**
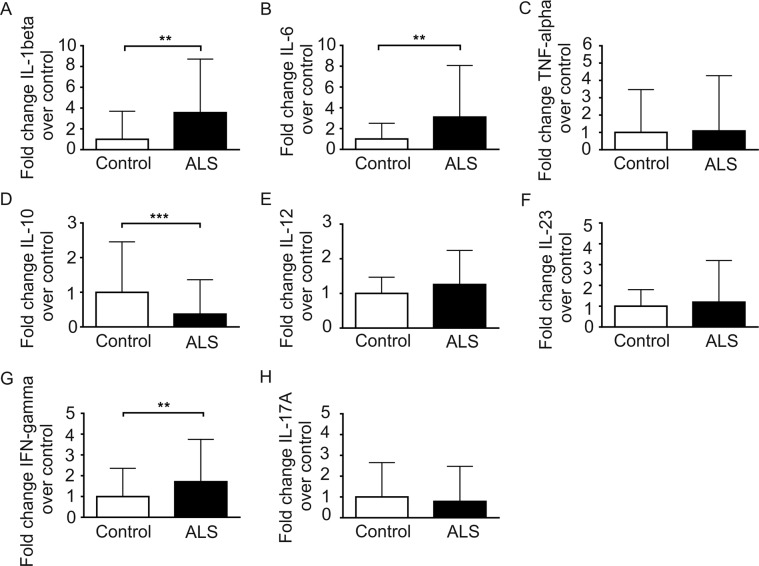
Cytokine profiles in serum of ALS patients and healthy controls. Levels of IL-1beta (**A**), IL-6 (**B**), TNF-alpha (**C**), IL-10 (**D**), IL-12 (**E**), IL-23 (**F**), IFN-gamma (**G**) and IL-17A (**H**) in the serum of ALS patients and healthy controls were calculated as fold change over the mean of healthy controls. Data are depicted as histograms (means ± SD). *p < 0.05, **p < 0.01, ***p < 0.001.

In order to study the cytokine profile in ALS, we analyzed pro-inflammatory cytokines (IL-1beta, IL-6, TNF-alpha), the anti-inflammatory cytokine IL-10 as well as cytokines that assist in T cell modulation and programming (IL-12 and IL-23) and that characterize specific T cell responses (IFN-gamma and IL-17A). In the ALS group, the level of IL-1 beta (p < 0.01), IL-6 (p < 0.01) and INF-gamma (p < 0.01) were significantly higher in comparison to controls whereas the anti-inflammatory cytokine IL-10 (p < 0.001) was significantly decreased (Fig. 4). There were no differences in serum cytokine expression of TNF-alpha, IL-12, Il-23 and IL-17A between ALS and controls (Fig. 4).

### Cytokine expression in relation to ALS progression rate

To study differences in the cytokine profile between slow and fast progressive ALS, we divided the total ALS group into fast and slow ALS as described above. We found that IL-1beta (p < 0.05) was significantly higher in fast compared to slow ALS (Suppl. Fig 5A). All other studied cytokines including IL-6, TNF-alpha, IL-10, IL-12, IL-23, IFN-gamma, and IL-17A showed no significant differences between fast and slow ALS (Suppl. Fig. 5B–H).

### Cytokine profile in correlation to clinical characteristics

Correlation analysis between cytokines and clinical/demographic parameters revealed no correlations to age, BMI, ppFVC and ALSFRS-R score. IL-6 (r = 0.515) positively correlated to disease duration. IL-1beta (r = 0.352) correlated positively to ALSFRS-R-slope (Suppl. Tab. 2).

### Immune cell phenotyping in CSF of ALS patients

In a subgroup of ALS patients (16) and controls (10) we studied immune cell subsets in CSF samples. Lymphocytes, CD3 + T cells, CD4 + T cells, CD8 + T cells, CD4 + naïve T cells, CD4 + memory T cells, CD8 + naïve T cells, CD8 + memory T cells, B cells, naïve B cells, memory B cells, NK cells, NKT cells, CD4 + NKT cells, CD8 + NKT cells, classical Mo, intermediate Mo, non-classical Mo, slanDCs and BDCA1 + DCs were evaluated by FACS analysis. By doing so, we found significantly higher amounts of lymphocytes and naïve CD8 + T cells in ALS patients’ CSF compared to controls’ CSF. All other cell types were not significantly different between the two groups (Table 2).

## Discussion

Emerging evidence suggests that neuroinflammation is involved in the degenerative process of MNs in ALS, indicating a vital role of the systemic immune system in the pathophysiology of ALS^31–33^. However, the impact of the immune system in ALS is not clear and contradictory results for the immune and cytokine profile in blood and CSF of ALS patients were recently published^16,18,19,21–23^. The aim of this project was to comprehensively study immunophenotypes in ALS patients and to determine whether these immunological changes correlate with diverse disease-related markers/phenotypes.

Total amounts of lymphocytes, T cells and APC cells were not different between ALS and controls. However, T cell subsets were shifted towards a pro-inflammatory immune response in peripheral blood of ALS patients. While CD4 + T cells were decreased, CD8 + T cells were found to be increased with a concomitant decrease of CD4/CD8 ratio. Previous studies showed contradictory findings with either no change of CD4 + T and CD8 + T cells^22^ or an increase in CD4 + T cells in combination with a decrease of CD8 + T cells^23^ or unchanged CD8 + T cells in ALS^34^. In line with previous studies, pro-inflammatory T cell subsets including Th1 and Th17 cells were increased while Th2 cells and Treg cells were reduced in peripheral blood of ALS patients^24–27^. Th1 cells and especially Th17 cells are important players in pro-inflammatory T cell-mediated responses initiating and perpetuating pro-inflammatory immune responses in different autoimmune diseases^35,36^. Within the immunoregulatory network the modulation of regulatory and inflammatory conditions are important to balance distinct immune responses. APC are important players to weigh pro-inflammation in contrast to anti-inflammation by specific T cell programming^37^. Interestingly, the ratios of Th1/Th2 cells as well as Treg/Th17 cells were skewed towards a pro-inflammatory phenotype in our ALS cohort considering a shifted balance that maintains neuroinflammation in ALS disease. Cell counts of APC and B cells were not changed in ALS patients compared to healthy controls. Evaluations of functional characteristics e.g. activation and maturation profiles, co-stimulatory molecule expression or differences in antigen-presentation were not included in our study, therefore further studies have to elucidate if differences in functional properties are linked to T cell regulatory potential and ALS disease.

In contrast to a previous study, NKT cells were not increased in our ALS cohort^21^. However, we found increased levels of NK cells which is consistent with recent studies^21,22,38^, with a shift to the CD56^bright^NK subtype and a significant decrease of the CD56^dim^NK subtype. It is believed that these both subtypes have distinct roles in the human immune response. CD56^bright^NK cells are poorly cytotoxic, have modulatory functions of the immune system and produce high levels of cytokines, especially IFN-gamma following activation by monocytes. In contrast, CD56^dim^NK cells have primarily cytotoxic behavior^39^. CD56^bright^ and CD56^dim^NK cell are both immunoregulatory and can kill CD4 + T cells^40^. However, CD56^dim^NK cells are predominantly found in peripheral blood and CD56^bright^NK in lymphoid tissue^39,41^. Therefore, it is surprising that a shift to CD56^bright^NK cells is present in our ALS patients. Studies investigating the role of NK cells in CNS of ALS patients are lacking, but it is conceivable that NK cells could penetrate the blood-brain barrier and interact with local APC like microglia and astroglia^42,43^. However, we did not find increased amounts of NK cells in the CSF of ALS patients, which might be due to the small sample size or because of the cells stick to CNS tissue and will therefore not be found in CSF. Interestingly however is the fact that in slowly progressive ALS modulatory rather than cytotoxic CD56^bright^NK cells were increased. In multiple sclerosis, reduced disease activity is observed during pregnancy, which could be linked to the increase in the percentage of CD56^bright^ regulatory NK cells^44,45^.

Further innate immune cells are also activated in our cohort of ALS patients with an increase of monocytes^22,46^. Microglia are resident monocytes of the CNS and are predominantly yolk sac–derived from early embryonic days^47^, but circulating bone marrow-derived monocytes can immigrate into CNS in neurodegenerative disorders^48^. The role of peripheral monocytes in ALS pathology is not clear, but recent evidence suggests a neuroprotective role of peripheral monocytes in the pathogenesis of ALS^20,23^.

The changes of the serum cytokine levels in ALS patients likewise lead to the conclusion of an impaired balance of Th cells and their activity. IL-10, an anti-inflammatory cytokine, which is secreted by Th2 and Treg cells to limit an excessive immune reaction, was significantly decreased in ALS patients. In contrast, the proinflammatory cytokines IL-1 beta, IL-6 expressed by APC and monocytes and the most important effector cytokine of Th1, IFN-gamma, were significantly increased which is consistent with previous studies^13,49^. TNF-alpha, a classical inflammatory cytokine was shown to be significantly increased in ALS mice and patients^12,50^. However, TNF-alpha as well as IL-12 and IL-23 were not significantly changed in our cohort which is different from recent studies^19,51^. Genetic ablation of TNF-alpha showed no improvement in the progression of disease in SOD1-G93A mice^52^, raising doubts about the contribution of TNF-alpha in the pathogenesis of ALS. Furthermore, we did not find increased levels of IL-17A in ALS patients, even though Th17 cells were increased in our ALS cohort^51,53^.

**Figure 5:**
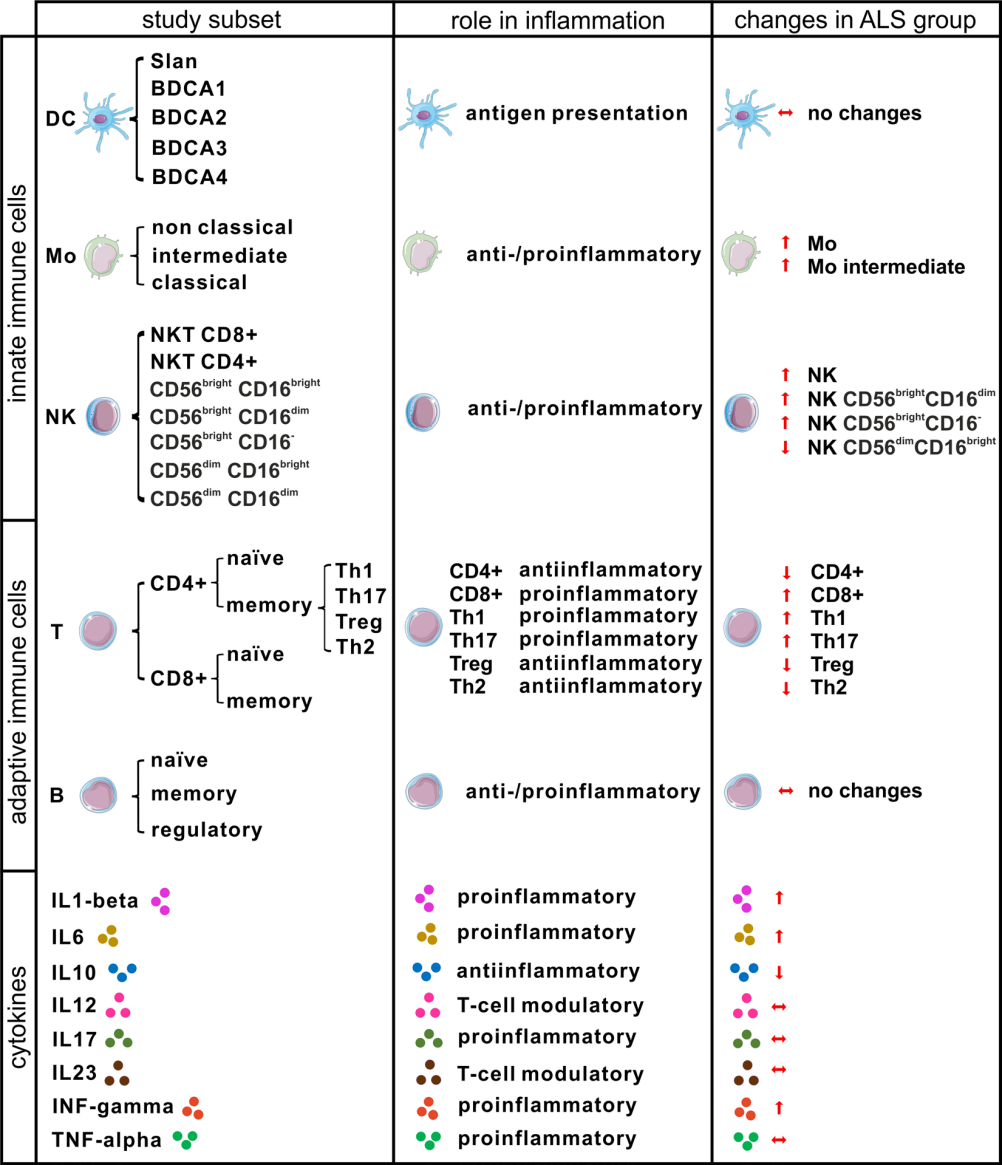
Summary of changes in the peripheral immune system in the ALS cohort. Cell icons of DC, Mo, NK, T and B cells were taken from https://smart.servier.com (provided by Les Laboratoires Servier) with Public Licence (Creative Commons Attribution 3.0 Unported License; https://creativecommons.org/licenses/by/3.0/).

In summary of all changes in immune cell types and cytokines we found, a significant shift of the peripheral immune system towards a proinflammatory cell-mediated immune response in ALS patients becomes obvious (Fig. 5). Furthermore, correlation analysis to clinical characteristics revealed that Th1 and Th17 cells increased with clinical severity (ALSFRS-R and ppFVC). Except for IL-1beta, which was significantly higher in fast ALS and CD56^bright^CD16^dim^NK cells, which were significantly higher in slow ALS, we found no significant differences between slow and fast progressive ALS regarding immune cells and cytokine levels. Therefore, it could be concluded that the proinflammatory Th1/Th17 shift develops with disease severity and disease progression.

Th1 and Th17 cells showed a weak negative correlation to respiratory function (ppFVC). Hypoxia and hypoxemia can strongly stimulate the immune system in ALS patients^54^, an activation of the immune systeme due to declining respiratory function can therefore not be excluded. However declining respiratory function also represents ALS disease progression^55^. It is therefore challenging to conclude whether the Th1/Th17 shift is a side effect following progressing neurodegeneration due to the accumulation of toxic products or a pathomechanistic e.g. autoimmune factor of disease progression or the reason of unspecific immune system activation due to e.g. aspiration with chronic pneumonia (mainly due to increasing respiratory deficiency and swallowing difficulties). Further studies with larger focus on the respiratory status and especially on hypoxia and hypoxemia are needed to clarify their influence on immune system activation in ALS disease.

Only a few neuropathological studies focused on the infiltration of peripheral immune cells in human CNS tissue of ALS patients, however a recent study showed increased amounts of CD8 + T cells in CNS tissue of ALS patients fitting to the hypothesis, that peripheral immune system activation might be a recruiting phenomenon for the CNS inflammation process^56^. The hypothesis of a double-edged sword of the immune system in ALS means a neuroprotective phase with increased anti-inflammatory cytokine release in very early or presymptomatic ALS disease. With increasing disease duration the role switches to a more aggressive, neurotoxic behavior with the release of cytotoxic cytokines^57–59^. Our study only shows a pro-inflammatory shift of the peripheral immune system in ALS, which increases with disease severity. This might depend on the fact, that the mean ALSFRS-R in our group was 32 points, which means relevant disability and therefore our patients were mainly in a progressed disease stage. Furthermore, our cross-sectional study design does not allow to answer this question properly. Longitudinal studies both in presymptomatic phases in gene carrier cohorts or studies of patients close to symptom onset are needed to investigate early anti-inflammatory modulation of the immune system to clarify this question.

Which therapeutic conclusions can be made on the finding, that the immune system is shifted to proinflammation in progressing disease stages of ALS? This question is not easy to answer, because we do not know yet which role does neuro/inflammation play in the pathophysiology of ALS. However, modulation of the immune system might be an easily accessible treatment target for ALS. Several drug developmental studies are focused on this topic. For instance, modulation of microglia activity with the oral treatment of Fasudil seems to be effective in preclinical studies and a clinical trial is currently ongoing^60–62^. Other human clinical trials with immune modulatory compounds have already shown positive effects while others failed^63–65^. A very recent small pilot study investigated autologous infusions of expanded Treg cells as a disease-modifying treatment in ALS patients and the authors show a potential benefit of the treatment^66^. Another recent study demonstrated a possible clinical benefit lasting at least 6 months with intrathecal administered bone-marrow mesenchymal stem cells (MSCs), which might influence neuroinflammation^67^. Interestingly enough, co-culture experiments of such MSCs with PBMCs of ALS patients showed a shift towards Treg/Th2 activation and expression of anti-inflammatory cytokines such as IL-10, which both are reduced in ALS patients (Figs. 1 and 4)^68^. However, due to the complexity of the immune system, a proper understanding of the dynamics and roles of each immune cell is necessary to design successful targeted treatments.

Further longitudinal studies on functional properties of specific immune cells especially including patients in early disease stages are needed to explore the interplay of peripheral innate and adaptive immunity in more detail and the relevance for inflammation in the CNS compartment through the course of the disease.

## Supplementary information

Supplementary information.
