# Supplementary material for: Peripheral proinflammatory Th1/Th17 immune cell shift is linked to disease severity in amyotrophic lateral sclerosis
Source: Sci Rep. 2020 Apr 3;10:5941. doi: 10.1038/s41598-020-62756-8 (PMC7125229; doi:10.1038/s41598-020-62756-8)
Supplement: Supplementary file 1 — Supplementary information. [file 41598_2020_62756_MOESM1_ESM.pdf]

SUPPLEMENTARY INFORMATION

Peripheral proinflammatory Th1/Th17 immune cell shift is linked to disease severity in amyotrophic lateral sclerosis

Authors:

Mengmeng Jin[1,2,#], Rene Günther[1,3,#,*], Katja Akgün[1,2], Andreas Hermann[+1,3-5], Tjalf Ziemssen[+1,2]

[#]Contribute equally as first author

[+]Contribute equally as senior author

* corresponding author

Supplementary Tables:

Supplementary Table1: Correlation of immune cells to clinical parameters in ALS

Immune cells	ALSFRS-R score	ALSFRS-R slope	disease duration	ppFVC	Age	BMI
**CD3+ T cells**	r = -0.17<br>p = 0.188<br>N = 64	r = 0.23<br>p = 0.071<br>N = 64	r = -0.13<br>p = 0.312<br>N = 66	r = -0.04<br>p = 0.766<br>N = 61	r = 0.09<br>p = 0.449<br>N = 66	r = -0.14<br>p = 0.270<br>N = 65
**CD4+ T cells**	r = 0.07<br>p = 0.563<br>N = 64	r = 0.005<br>p = 0.966<br>N = 64	r = -0.10<br>p = 0.41<br>N = 66	r = 0.06<br>p = 0.638<br>N = 61	r = -0.14<br>p = 0.24<br>N = 66	r = 0.002<br>p = 0.987<br>N = 65
**CD8+T cells**	r = 0.07<br>p = 0.582<br>N = 64	r = -0.07<br>p = 0.567<br>N = 64	r = 0.02<br>p = 0.900<br>N = 66	r = 0.18<br>p = 0.161<br>N = 61	**r = -0.36**<br>**p = 0.003**<br>**N = 66**	r = 0.04<br>p = 0.759<br>N = 65
**CD4/CD8**	r = 0.04<br>p = 0.726<br>N = 64	r = 0.08<br>p = 0.518<br>N = 63	r = 0.08<br>p = 0.543<br>N = 64	r = -0.25<br>p = 0.062<br>N = 59	r = 0.14<br>p = 0.260<br>N = 66	r = -0.13<br>p = 0.312<br>N = 63
**Th1 cells**	**r = - 0.45**<br>**p = 0.002**<br>**N = 64**	**r = 0.29**<br>**p = 0.021**<br>**N = 64**	r = 0.16<br>p = 0.206<br>N = 66	**r = -0.26**<br>**p = 0.045**<br>**N = 61**	r = -0.21<br>p = 0.101<br>N = 66	r = 0.05<br>p = 0.708<br>N = 65
**Th2 cells**	r = 0.05<br>p = 0.722<br>N = 64	r = -0.03<br>p = 0.800<br>N = 64	r = 0.05<br>p = 0.690<br>N = 66	r = -0.12<br>p = 0.365<br>N = 61	r = -0.03<br>p = 0.820<br>N = 66	r = 0.02<br>p = 0.885<br>N = 65
**Th1/Th2**	r = -0.13<br>p = 0.291<br>N = 64	**r = 0.31**<br>**p = 0.0114**<br>**N = 64**	r = -0.16<br>p = 0.170<br>N = 63	r = -0.0045<br>p = 0.974<br>N = 57	r = -0.07<br>p = 0.581<br>N = 63	r = -0.04<br>p = 0.775<br>N = 62

**Treg cells**	r = -0.03<br>p = 0.802<br>N = 65	r = 0.02<br>p = 0.860<br>N = 65	r = -0.07<br>p = 0.600<br>N = 67	r = -0.01<br>p = 0.365<br>N = 62	r = -0.07<br>p = 0.600<br>N = 67	r = 0.07<br>p = 0.583<br>N = 66
**Th17 cells**	**r = -0.41**<br>**p = 0.00089**<br>**N = 64**	r = 0.06<br>p = 0.660<br>N = 64	**r = 0.34**<br>**p = 0.006**<br>**N = 66**	**r = -0.30**<br>**p = 0.018**<br>**N = 61**	r = -0.06<br>p = 0.605<br>N = 66	r = -0.12<br>p = 0.345<br>N = 65
**Treg/Th17**	**r = 0.29**<br>**p = 0.020**<br>**N = 64**	r = -0.10<br>p = 0.434<br>N = 64	r = -0.03<br>p = 0.785<br>N = 65	r = 0.22<br>p = 0.097<br>N = 61	r = -0.11<br>p = 0.390<br>N = 65	r = -0.02<br>p = 0.886<br>N = 64
**NK cells**	r = 0.09<br>p = 0.464<br>N = 64	r = -0.03<br>p = 0.800<br>N = 64	r = -0.05<br>p = 0.700<br>N = 66	r = -0.04<br>p = 0.745<br>N = 61	r = 0.00071<br>p = 1.000<br>N = 66	r = 0.06<br>p = 0.626<br>N = 65
**CD56^{bright}**<br>**CD16^{bright}**<br>**NK cells**	r = -0.02<br>p = 0.890<br>N = 64	r = -0.01<br>p = 0.965<br>N = 64	r = 0.03<br>p = 0.812<br>N = 66	r = 0.07<br>p = 0.588<br>N = 61	r = 0.06<br>p = 0.660<br>N = 66	r = -0.15<br>p = 0.249<br>N = 65
**CD56^{bright}**<br>**CD16^{dim}**<br>**NK cells**	r = 0.05<br>p = 0.720<br>N = 64	r = -0.13<br>p = 0.298<br>N = 64	r = 0.10<br>p = 0.422<br>N = 66	r = 0.13<br>p = 0.305<br>N = 61	r = -0.22<br>p = 0.072<br>N = 66	r = 0.11<br>p = 0.400<br>N = 65
**CD56^{bright}**<br>**CD16-**<br>**NK cells**	r = 0.02<br>p = 0.900<br>N = 64	r = -0.09<br>p = 0.465<br>N = 64	r = 0.12<br>p = 0.333<br>N = 66	r = 0.09<br>p = 0.504<br>N = 61	r = -0.16<br>p = 0.189<br>N = 66	r = 0.01<br>p = 0.960<br>N = 65
**CD56^{dim}**<br>**CD16^{dim}**<br>**NK cells**	r = 0.07<br>p = 0.607<br>N = 64	r = 0.09<br>p = 0.472<br>N = 64	r = -0.17<br>p = 0.173<br>N = 66	r = -0.05<br>p = 0.703<br>N = 61	r = -0.08<br>p = 0.500<br>N = 66	r = 0.09<br>p = 0.498<br>N = 65
**CD56^{dim}**<br>**CD16^{bright}**<br>**NK cells**	r = -0.01<br>p = 0.925<br>N = 64	r = 0.05<br>p = 0.705<br>N = 64	r = -0.06<br>p = 0.650<br>N = 66	r = -0.12<br>p = 0.379<br>N = 61	**r = 0.39**<br>**p = 0.001**<br>**N = 66**	r = -0.12<br>p = 0.346<br>N = 65

**NKT cells**	r = 0.14<br>p = 0.263<br>N = 64	r = 0.002<br>p = 0.987<br>N = 64	r = -0.18<br>p = 0.141<br>N = 66	r = 0.07<br>p = 0.612<br>N = 61	r = -0.18<br>p = 0.143<br>N = 66	r = 0.09<br>p = 0.497<br>N = 65
**CD4+NKT cells**	r = -0.05<br>p = 0.691<br>N = 64	r = 0.02<br>p = 0.893<br>N = 64	r = -0.04<br>p = 0.776<br>N = 66	r = -0.02<br>p = 0.890<br>N = 61	r = 0.02<br>p = 0.842<br>N = 66	r = -0.11<br>p = 0.387<br>N = 65
**CD8+NKT cells**	r = 0.01<br>p = 0.938<br>N = 64	r = 0.06<br>p = 0.628<br>N = 64	r = -0.16<br>p = 0.194<br>N = 66	r = -0.15<br>p = 0.256<br>N = 61	r = -0.07<br>p = 0.590<br>N = 66	r = -0.03<br>p = 0.801<br>N = 65
**CD14+Mo**	r = 0.14<br>p = 0.287<br>N = 63	r = -0.11<br>p = 0.403<br>N = 63	r = -0.13<br>p = 0.322<br>N = 65	r = 0.02<br>p = 0.868<br>N = 60	r = -0.14<br>p = 0.260<br>N = 65	r = -0.05<br>p = 0.711<br>N = 65
**Classical Mo**	r = 0.15<br>p = 0.256<br>N = 63	r = -0.01<br>p = 0.920<br>N = 63	r = -0.05<br>p = 0.670<br>N = 65	r = 0.03<br>p = 0.827<br>N = 60	r = 0.03<br>p = 0.810<br>N = 65	r = -0.02<br>p = 0.895<br>N = 64
**Intermediate Mo**	r = -0.01<br>p = 0.928<br>N = 63	r = -0.02<br>p = 0.890<br>N = 63	r = 0.04<br>p = 0.750<br>N = 65	r = 0.07<br>p = 0.582<br>N = 60	r = -0.04<br>p = 0.760<br>N = 65	r = -0.04<br>p = 0.744<br>N = 64
**Non-classical Mo**	r = -0.04<br>p = 0.762<br>N = 63	r = -0.07<br>p = 0.590<br>N = 63	r = -0.0024<br>p = 0.900<br>N = 65	r = 0.09<br>p = 0.496<br>N = 60	r = -0.05<br>p = 0.700<br>N = 65	r = 0.10<br>p = 0.443<br>N = 64

Supplementary Table 2: Correlation between cytokines in serum and clinical parameters in ALS

Cytokines in the Serum	ALSFRS-R score	ALSFRS-R slope	disease duration	ppFVC	Age	BMI
**IL-6**	r = 0.11<br>p = 0.349<br>N = 71	r = -0.13<br>p = 0.272<br>N = 70	**r = 0.52**<br>**p < 0.0001**<br>**N = 71**	r = 0.03<br>p = 0.821<br>N = 65	r = 0.07<br>p = 0.550<br>N = 71	r = 0.03<br>p = 0.789<br>N = 70
**IL-1beta**	r = -0.03<br>p = 0.79<br>N = 71	**r = 0.35**<br>**p = 0.004**<br>**N = 70**	r = 0.03<br>p = 0.831<br>N = 71	r = -0.16<br>p = 0.205<br>N = 65	r = -0.13<br>p = 0.278<br>N = 71	r = -0.01<br>p = 0.3<br>N = 70
**IFN-gamma**	r = 0.086<br>p = 0.476<br>N = 71	r = -0.0492<br>p = 0.686<br>N = 70	r = 0.16<br>p = 0.184<br>N = 71	r = -0.10<br>p = 0.446<br>N = 65	r = 0.003<br>p = 0.980<br>N = 71	r = -0.10<br>p = 0.417<br>N = 70

Supplementary Figures

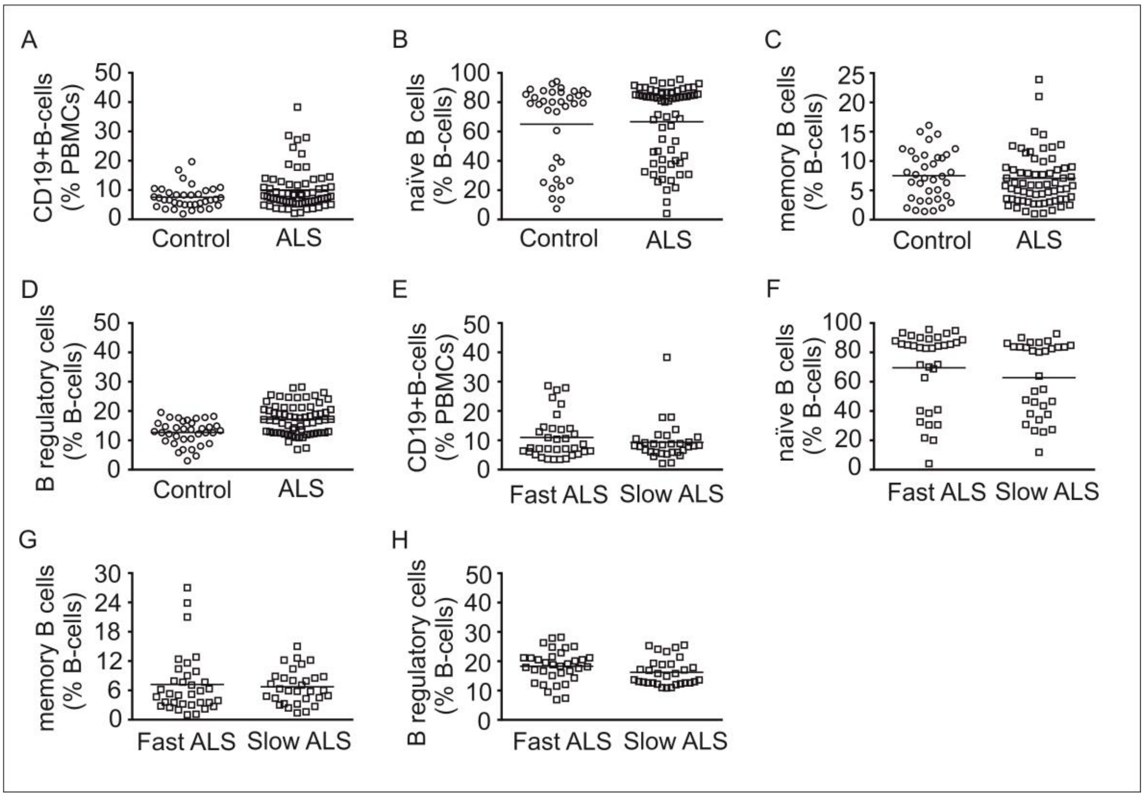

Supplementary Figure 1 Peripheral B cell profiles in ALS patients and healthy controls.

Depicted are relative counts of CD19+ B cells (**A**), naïve B cells (**B**), memory B cells (**C**) and B regulatory cells (**D**) of ALS versus controls as well as relative counts of CD19+ B cells (**E**), naïve B cells (**F**), memory B cells (**G**) and B regulatory cells (**H**) of fast ALS versus slow ALS. Results are presented as raw data, each circle represents one healthy control and each quad represents one ALS patient, horizontal lines indicate mean values. *p < 0.05, **p < 0.01, ***p < 0.001.

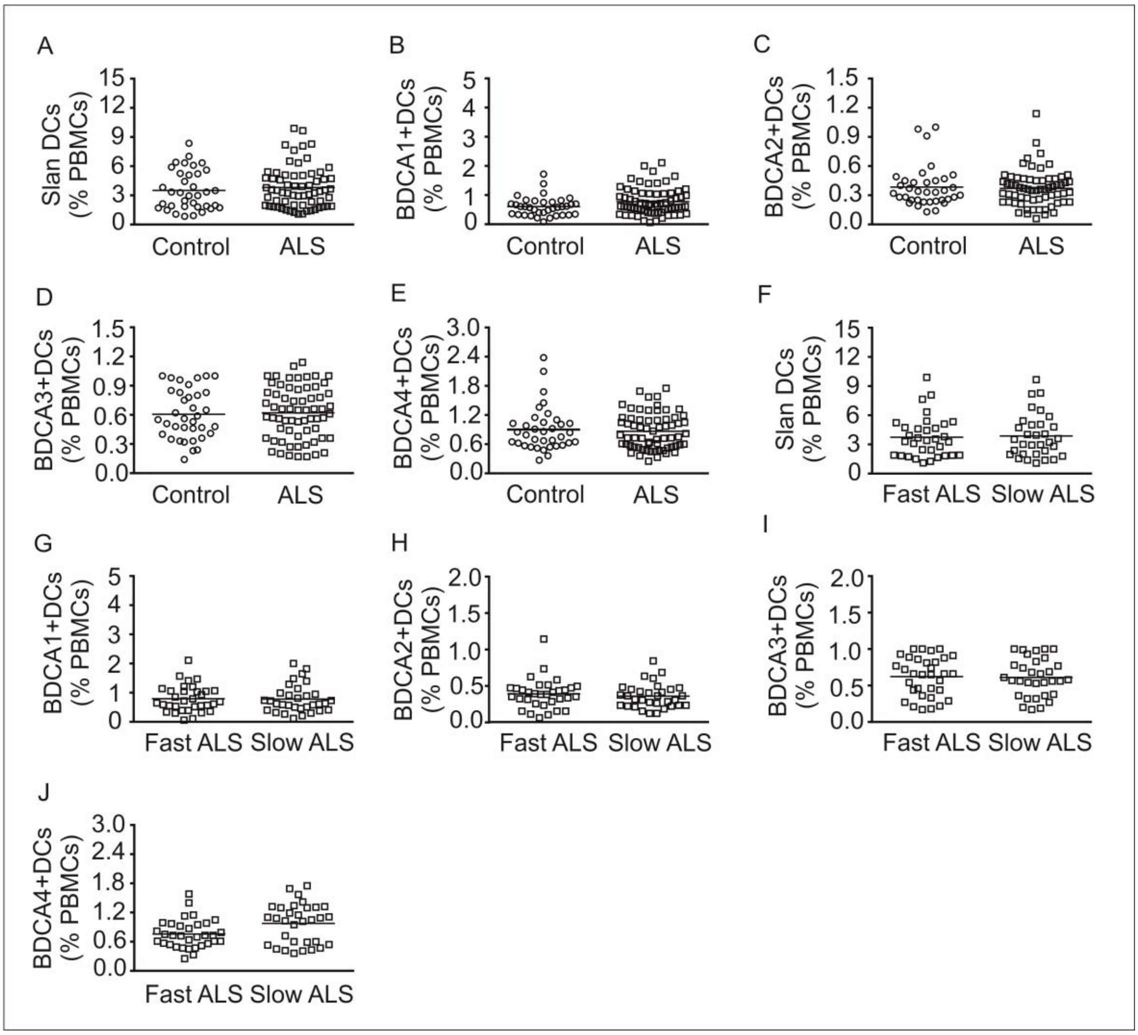

Supplementary Figure 2 Peripheral DC subsets in ALS patients and healthy controls.

Depicted are relative counts of slanDCs (**A**), BDCA1+DCs (**B**), BDCA2+DCs (**C**), BDCA3+DCs (**D**) and BDCA4+DCs (**E**) of ALS versus controls as well as relative counts of SlanDCs (**F**), BDCA1+DCs (**G**), BDCA2+DCs (**H**), BDCA3+DCs (**I**) and BDCA4+DCs (**J**) of fast ALS versus slow ALS. Results are presented as raw data, each circle represents one healthy control and each quad represents one ALS patient, horizontal lines indicate mean values. *p < 0.05, **p < 0.01, ***p < 0.001.

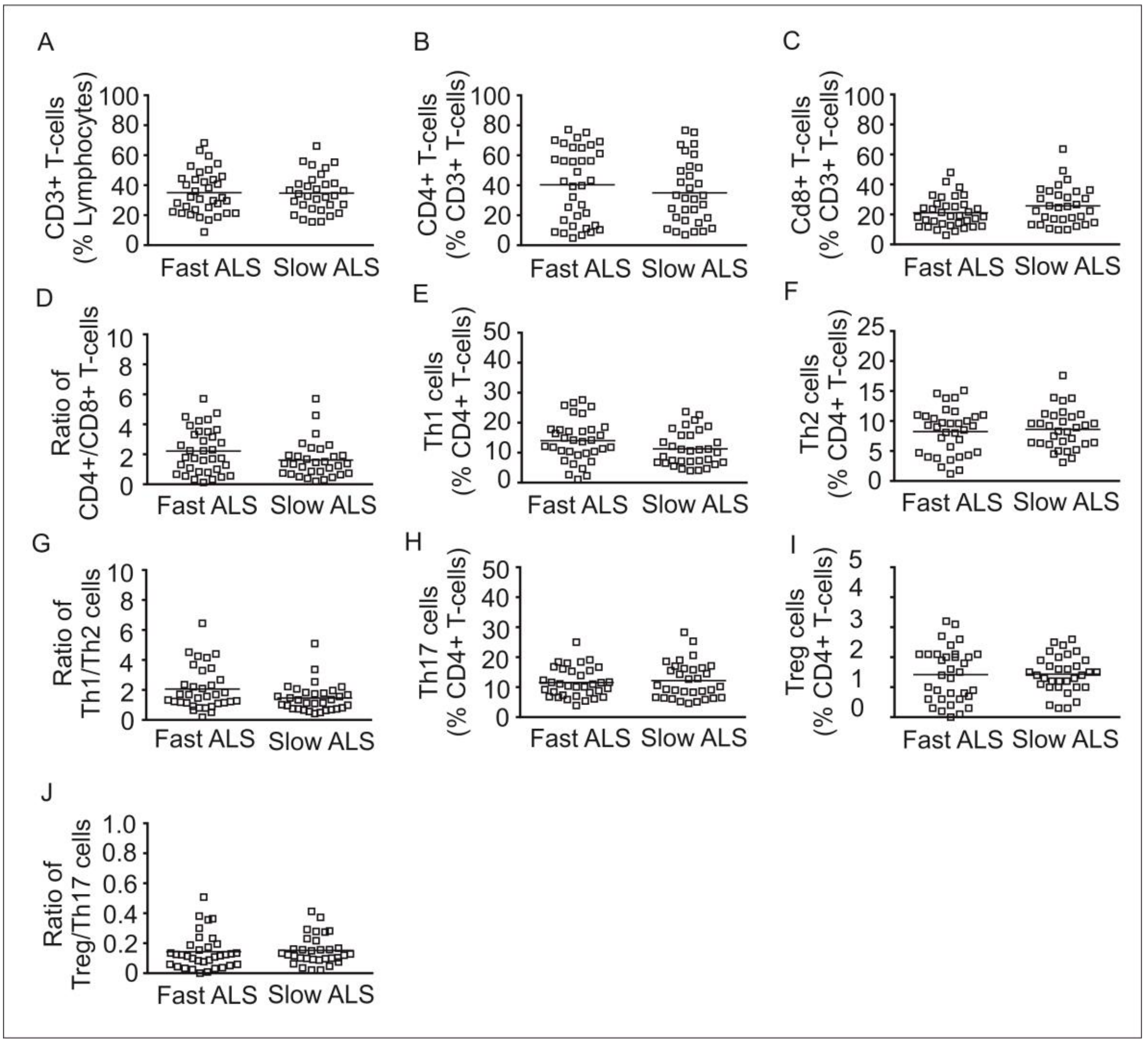

Supplementary Figure 3 Peripheral T cell and B cell subsets in comparison of fast versus slow ALS. Depicted are relative counts of CD3+T cells (**A**), CD4+T cells (**B**), CD8+T cells (**C**), ratio of CD4+/CD8+T cells (**D**), Th1 cells (**E**), Th2 cells (**F**), ratio of Th1/Th2 cells (**G**), Th17 cells (**H**) and Treg cells (**I**) and the ratio of Treg/Th17 cells (**J**) of fast ALS versus slow ALS. Results are presented as raw data, each circle represents one healthy control and each quad represents one ALS patient, horizontal lines indicate mean values. *p < 0.05, **p < 0.01, ***p < 0.001.

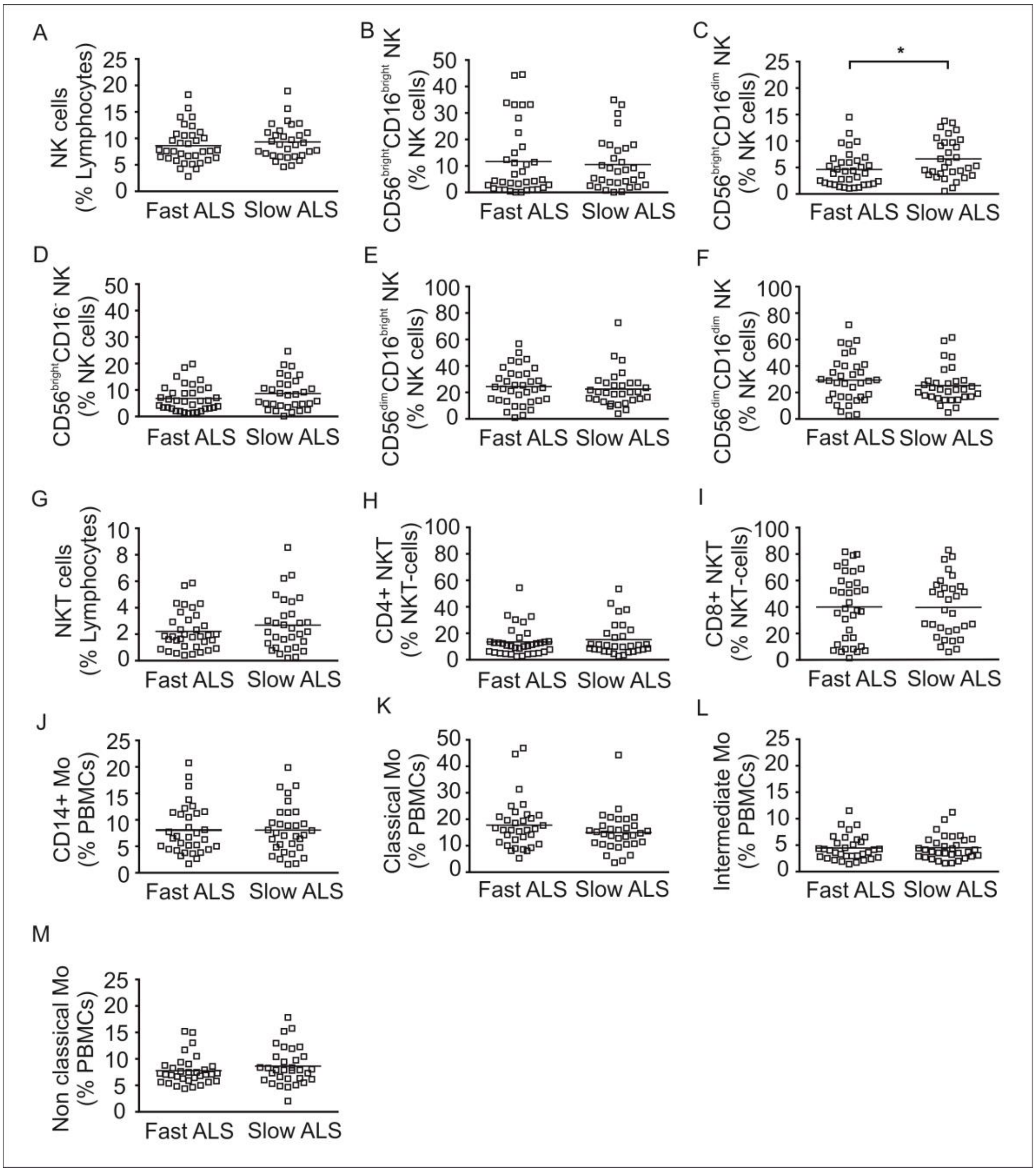

Supplementary Figure 4 Peripheral NK cells and Mo subset of fast versus slow ALS. Depicted are relative counts of NK cells (**A**), $CD56^{bright}CD16^{bright}$NK cells (**B**), $CD56^{bright}CD16^{dim}$NK cells (**C**), $CD56^{bright}CD16^{-}$NK cells (**D**), $CD56^{dim}CD16^{bright}$NK cells (**E**), $CD56^{dim}CD16^{dim}$NK cells (**F**), NKT cells (**G**), CD4+NKT cells (**H**), CD8+NKT cells (**I**), CD14+Mo (**J**), classical Mo cells (**K**), intermediate Mo cells (**L**), and non-classical Mo cells (**M**) of fast versus slow ALS. Results are presented as raw data, each circle represents one healthy control and each quad represents one ALS patient, horizontal lines indicate mean values. *$p < 0.05$, **$p < 0.01$, ***$p < 0.001$.

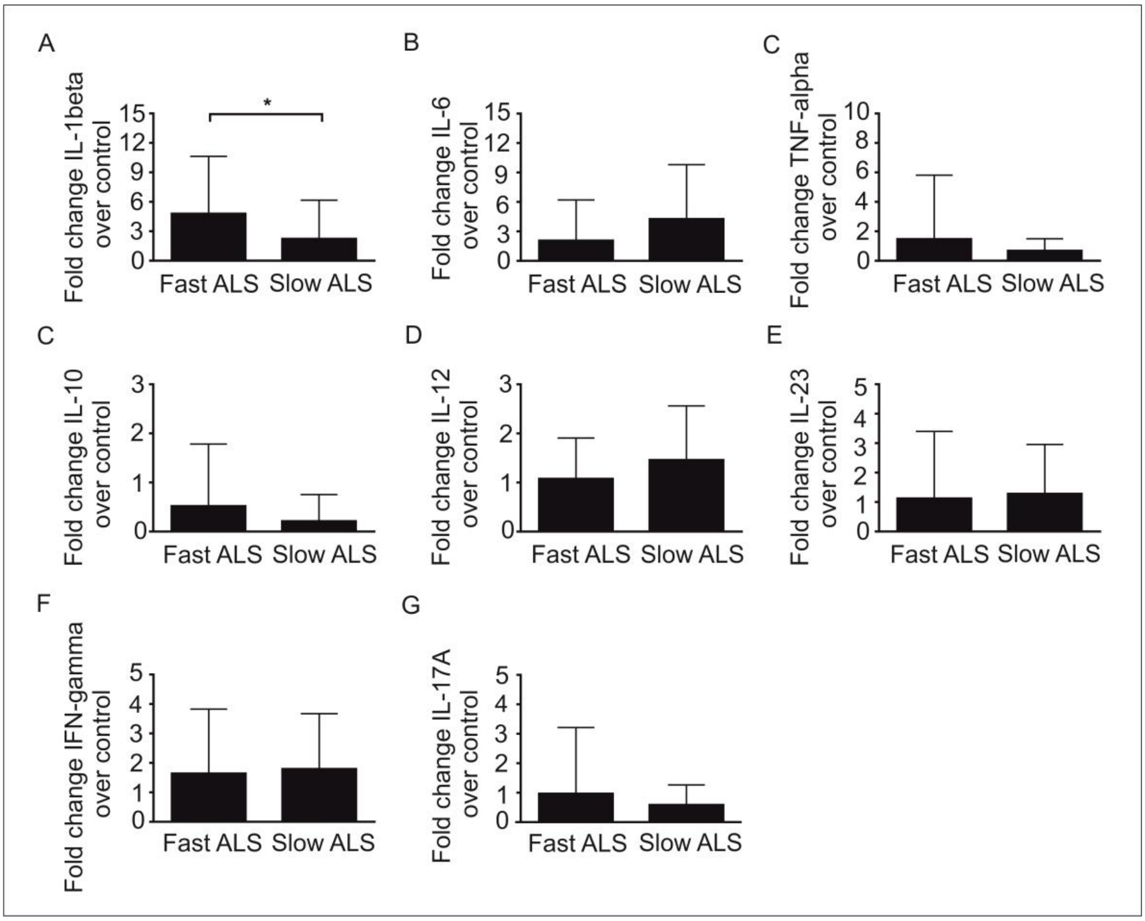

Supplementary Figure 5 Cytokines in the blood serum of fast versus slow ALS. Levels of IL-1beta (**A**), IL-6 (**B**), TNF-alpha (**C**), IL-10 (**D**), IL-12 (**E**), IL-23 (**F**), IFN-gamma (**G**) and IL-17A (**H**) in the serum of fast ALS and slow ALS were calculated as fold change over the mean of healthy controls. Data are depicted as histograms (means ± SD). *p < 0.05.

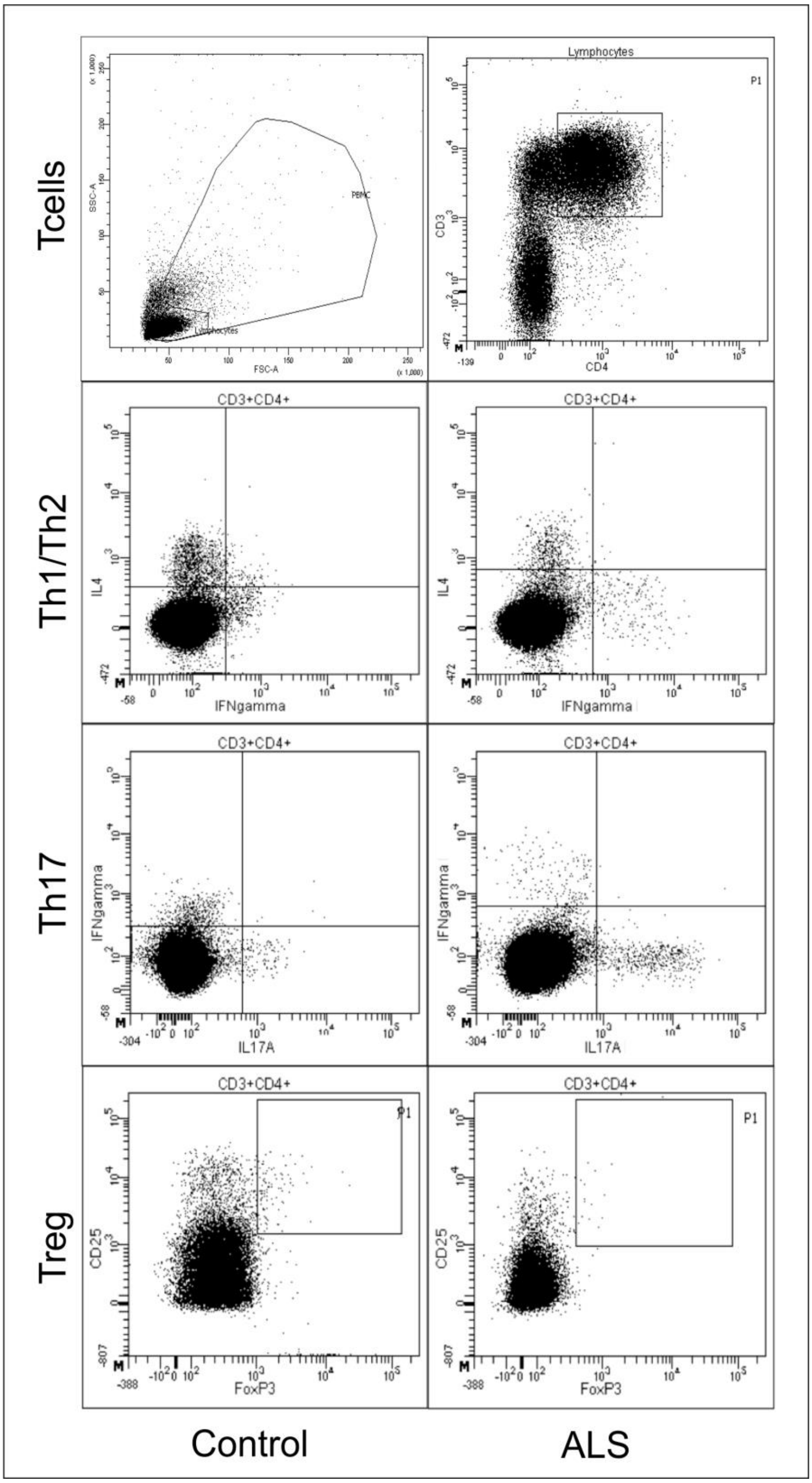

Supplementary Figure 6 Representative Facs plots of Tcells, Th1, Th17 and Treg cells in ALS patients and healthy controls.
